# Application for Burns

**Published:** 1893-06

**Authors:** 


### Application for Burns.

℞ Linseed oil, - - - ℥iv.

Lime-water, - - - ℥iv.

Thymol, - - - - grs. vj.

Dissolve the thymol in the oil before adding the lime-water.

